# Case Report: Prostatic infarction mimicking prostate cancer in a patient with markedly elevated prostate-specific antigen

**DOI:** 10.3389/fmed.2026.1959980

**Published:** 2026-09-30

**Authors:** Huajian Ye, Yaokang Chen, Liming Yu

**Affiliations:** Department of Urology, Shaoxing Second Hospital, Shaoxing, China

**Keywords:** case report, contrast-enhanced ultrasound, prostate cancer, prostate-specific antigen, prostatic infarction

## Abstract

Prostatic infarction is an uncommon benign ischemic lesion that may closely mimic prostate cancer through marked prostate-specific antigen (PSA) elevation and focal prostate abnormalities. A 69-year-old man with long-standing hypertension presented with progressive lower urinary tract symptoms and urinary retention. Pre-catheterization postvoid residual urine volume was approximately 430 mL. Total PSA was 31.858 ng/mL, free PSA was 1.291 ng/mL, and the free-to-total PSA ratio was 0.04. Magnetic resonance imaging revealed a well-circumscribed left transition-zone nodule with a Prostate Imaging Reporting and Data System (PI-RADS) score of 1, whereas contrast-enhanced ultrasound demonstrated a focal perfusion defect. Transperineal systematic biopsy with CEUS-guided targeted sampling and subsequent transurethral resection confirmed focal prostatic infarction with benign prostatic hyperplasia and basal cell hyperplasia, without adenocarcinoma. PSA decreased to 2.34 ng/mL at 1 month, remained low at 2.45 ng/mL at 3 months, and was 1.234 ng/mL approximately 16 months after TURP. This case highlights that prostatic infarction, although uncommon, may contribute to marked PSA elevation and mimic prostate cancer. In patients with high PSA levels and discordant biochemical and imaging findings, prostatic infarction should be considered as a benign differential diagnosis alongside BPH, prostatitis, prostatic abscess, and other non-malignant causes.

## Introduction

Prostatic infarction is an uncommon benign ischemic lesion that is usually recognized in biopsy, surgical, or autopsy specimens rather than before histopathological examination (1). Previous pathological series have shown that prostatic infarction is only rarely identified in routine prostate needle biopsy specimens (1). Its clinical importance lies in its ability to mimic prostate cancer. Patients may present with urinary retention or hematuria, and serum PSA can rise markedly (1, 2). In this setting, prostate cancer, severe prostatitis, abscess, or other benign mimickers may be considered (3).

The mechanism is likely multifactorial. Atherosclerosis, embolic events, local trauma, and prolonged immobilization have been proposed as possible contributors (4). Vascular disease may impair prostatic arterial and microvascular perfusion and increase susceptibility to ischemic injury. Benign prostatic hyperplasia (BPH) may further predispose the prostate to ischemia through progressive nodular enlargement, which can increase intraprostatic tissue pressure, compress small intraprostatic vessels, and distort or stretch the local vascular architecture. These changes may reduce microvascular perfusion and oxygen delivery to the enlarged gland (5, 6, 15). Acute urinary retention may coexist with prostatic infarction and may reflect substantial local obstructive and hemodynamic disturbance (1, 15). The free-to-total PSA ratio is commonly used as an adjunctive parameter in prostate cancer risk assessment within selected PSA ranges; however, its interpretation becomes less certain at markedly elevated total PSA levels (7). We report a diagnostically challenging case of focal prostatic infarction in an elderly man who presented with urinary retention, markedly elevated PSA, and a very low free-to-total PSA ratio, prompting clinical concern for prostate cancer despite the uncertain interpretation of the ratio at this PSA level. Although benign conditions such as BPH, prostatitis, and prostatic abscess are commonly considered when malignancy is not confirmed, this case illustrates that prostatic infarction, an uncommon benign ischemic lesion, should also be considered in the differential diagnosis of marked PSA elevation.

## Case description

A 69-year-old man presented to our department with progressive lower urinary tract symptoms for more than one year, with worsening during the preceding week. He described urinary hesitancy, prolonged voiding time, urinary intermittency, frequency, and painful urination. He denied gross hematuria, fever, nausea, vomiting, flank pain, decreased urine output, or lower-extremity edema. There was no documented history of prior prostatic surgery, catheter-related trauma, ischemic heart disease, atrial fibrillation, or family history of prostate cancer.

Before admission, he had not received regular treatment for lower urinary tract symptoms and had not taken prostate-related medications. Because of acute urinary retention, the postvoid residual urine volume was estimated at approximately 430 mL, and an indwelling urethral catheter was placed on March 31, 2025. Formal uroflowmetry was not performed because of catheterization, and an International Prostate Symptom Score was not documented. His medical history was notable for hypertension for more than 20 years, controlled with irbesartan/hydrochlorothiazide and sustained-release nifedipine. He reported consuming approximately 500 mL of spirits daily for 40 years and denied smoking. Laboratory testing showed elevated alanine aminotransferase (ALT) of 98 U/L and aspartate aminotransferase (AST) of 52 U/L; however, no definite diagnosis of alcohol-related liver disease had been documented.

Digital rectal examination revealed an enlarged prostate with soft consistency and obliteration of the median sulcus, without tenderness, palpable nodule, or suspicious induration. Ultrasonography performed on March 31, 2025, showed prostatic enlargement with calcifications, measuring 61 × 46 × 53 mm, corresponding to an estimated prostate volume of approximately 77 mL using the ellipsoid formula (Figure 1A). This case report was prepared in accordance with the CARE guidelines.

**Figure 1:**
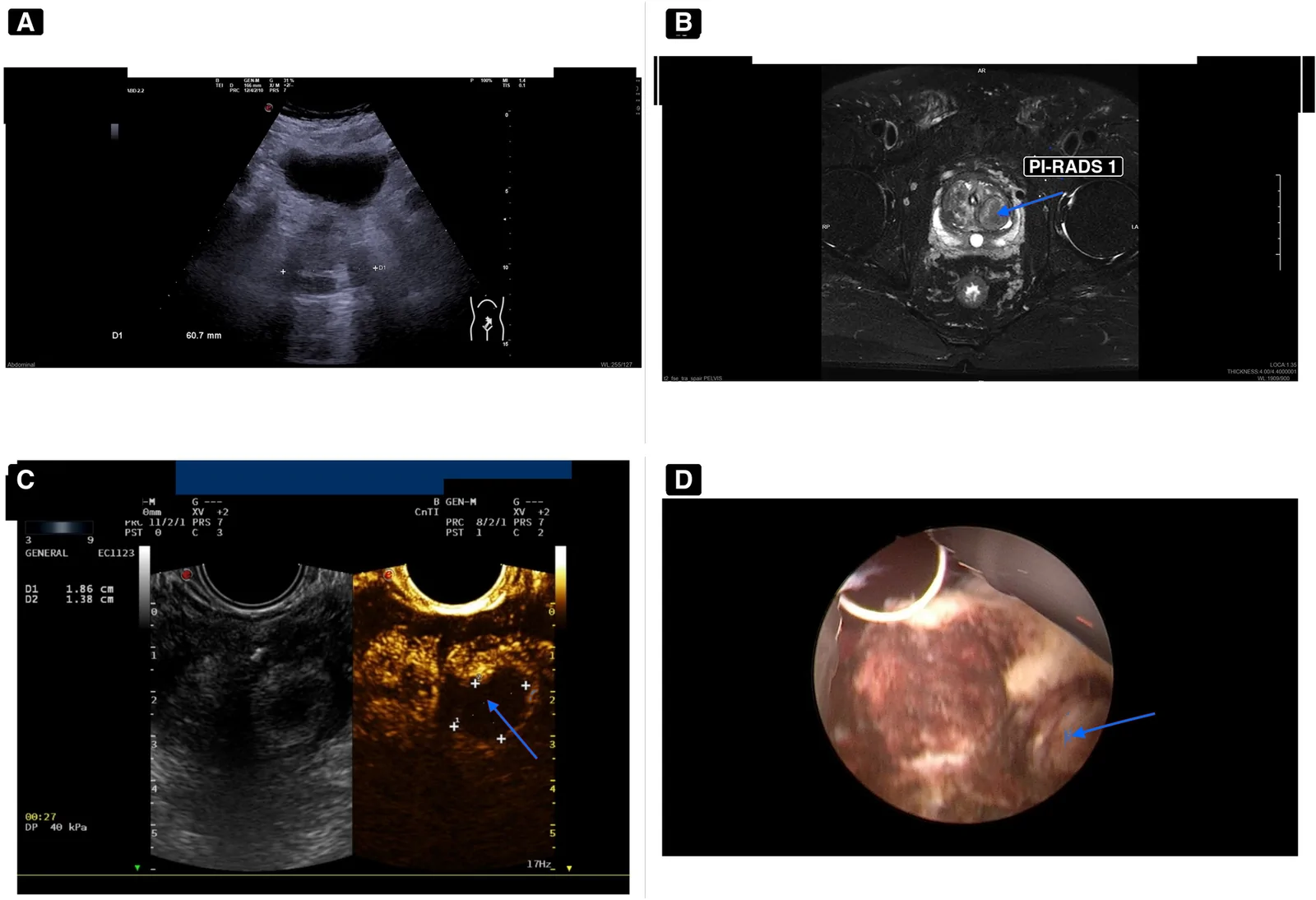
Imaging and intraoperative findings. (**A**) Ultrasonography showing prostatic enlargement with calcifications. (**B**) Magnetic resonance imaging showing a well-circumscribed left transition-zone nodule with a PI-RADS score of 1. (**C**) Contrast-enhanced ultrasound showing a focal perfusion defect in the left inner gland (arrow). (**D**) Intraoperative TURP view showing a purple-red nodular lesion with well-defined margins.

## Timeline

The diagnostic and therapeutic course is summarized in Table 1.

**Table 1:** Timeline of the diagnostic and therapeutic course.

Date/time	Event
March 31, 2025	Acute urinary retention; pre-catheterization PVR approximately 430 mL Indwelling urethral catheter placed Ultrasonography: prostatic enlargement with calcifications
April 6, 2025	PSA measured approximately 6 days after catheterization and before biopsy or surgery Total PSA: 31.858 ng/mL Free PSA: 1.291 ng/mL Free-to-total PSA ratio: 0.04
April 8, 2025	MRI: 19 × 20 mm left transition-zone nodule, PI-RADS 1 Pre-biopsy CEUS: focal non-perfused area in the left inner gland
April 8, 2025	Transperineal prostate biopsy performed 12 systematic cores obtained 2 CEUS-guided targeted cores obtained from the pre-existing perfusion abnormality
April 11, 2025	TURP performed for persistent LUTS and urinary retention Purple-red nodular lesion identified in the left prostatic lobe
After TURP	Pathology: BPH with focal basal cell hyperplasia and infarction No malignancy identified
1 month after TURP	Total PSA: 2.34 ng/mL Free PSA: 1.23 ng/mL Free-to-total PSA ratio: 0.53
3 months after TURP	Total PSA: 2.45 ng/mL Free PSA: 1.25 ng/mL Free-to-total PSA ratio: 0.51
August 10, 2026	Total PSA: 1.234 ng/mL Free PSA: 0.421 ng/mL Free-to-total PSA ratio: 0.34

## Diagnostic assessment

Serum PSA was measured on April 6, 2025, approximately 6 days after urethral catheterization and before prostate biopsy or surgery. Total PSA was 31.858 ng/mL and free PSA was 1.291 ng/mL, yielding a free-to-total PSA ratio of 0.04. Serum total and free PSA were measured using an Abbott ARCHITECT i2000 immunoassay system with a direct acridinium ester chemiluminescent immunoassay method. Although the free-to-total PSA ratio was very low (0.04), its diagnostic significance at a total PSA level of 31.858 ng/mL is uncertain because this value lies outside the range in which the ratio has been most extensively validated. Nevertheless, the overall PSA profile contributed to the clinical concern for malignancy (7).

MRI performed on April 8, 2025, demonstrated marked prostatic enlargement, measuring approximately 38 × 52 × 55 mm, with prominent enlargement and heterogeneous signal intensity of the transition zone. A well-circumscribed, encapsulated nodule measuring approximately 19 × 20 mm was identified in the left transition zone. The lesion showed no abnormal high signal on diffusion-weighted imaging and demonstrated peripheral enhancement after contrast administration. These findings were considered non-suspicious, with a PI-RADS score of 1 (Figure 1B) (8). The seminal vesicles were symmetric without abnormal signal, and no enlarged pelvic lymph nodes were identified. Quantitative apparent diffusion coefficient values were not reported in the original MRI examination. Although a PI-RADS score of 1 indicates a very low likelihood of clinically significant prostate cancer, the markedly elevated PSA level and symptomatic progression warranted further evaluation.

CEUS was performed before prostate biopsy on April 8, 2025, using a MyLab 8 ultrasound system equipped with an EC1123 transducer in contrast-tuned imaging (CNTI) mode. SonoVue microbubble contrast agent (2.4 mL) was administered intravenously, followed by a 5-mL normal saline flush. At approximately 19 s after contrast injection, enhancement was first observed in the bilateral inner glands. A non-perfused area measuring approximately 18.6 × 13.8 mm was identified in the left inner gland, whereas the right inner gland showed heterogeneous enhancement. At approximately 28 s, enhancement of the bilateral outer glands became apparent, with the boundary between the inner and outer glands remaining relatively clear. No asymmetric vascular architecture was observed during the early enhancement phase (Figure 1C). Quantitative CEUS parameters, including time-to-peak and formal wash-in/wash-out measurements, were not recorded; therefore, the CEUS assessment was qualitative.

This pre-existing focal perfusion abnormality was subsequently targeted during transperineal biopsy. The location was anatomically concordant with the focal infarction identified on biopsy and later confirmed in the TURP specimen. Although CEUS provided useful functional and localization information, the perfusion abnormality was non-specific and required histopathological confirmation (9). The differential diagnosis included focal prostatic infarction, prostatic abscess, granulomatous inflammation, and atypical prostate cancer.

Following MRI and CEUS, the patient underwent a transperineal prostate biopsy with CEUS-guided targeted sampling on April 8, 2025. A total of 14 biopsy cores were obtained, including 12 systematic cores, with six cores from each prostatic lobe, and two additional targeted cores from the left-sided nodule corresponding to the CEUS-defined perfusion-defect area. Histopathological examination of the biopsy specimens revealed BPH with focal infarction and basal cell hyperplasia (Figure 2A). Immunohistochemistry of the biopsy specimens showed PSA and prostate-specific acid phosphatase positivity, P504s negativity, preserved basal cells highlighted by CK5/6 and P63, and a low Ki-67 labeling index of approximately 2%; representative P63 staining is shown in Figure 2D. P504s negativity and preserved basal cells highlighted by CK5/6 and P63 argued against prostatic adenocarcinoma (10). Together with the histomorphological findings, these results were consistent with a benign ischemic lesion involving hyperplastic tissue.

**Figure 2:**
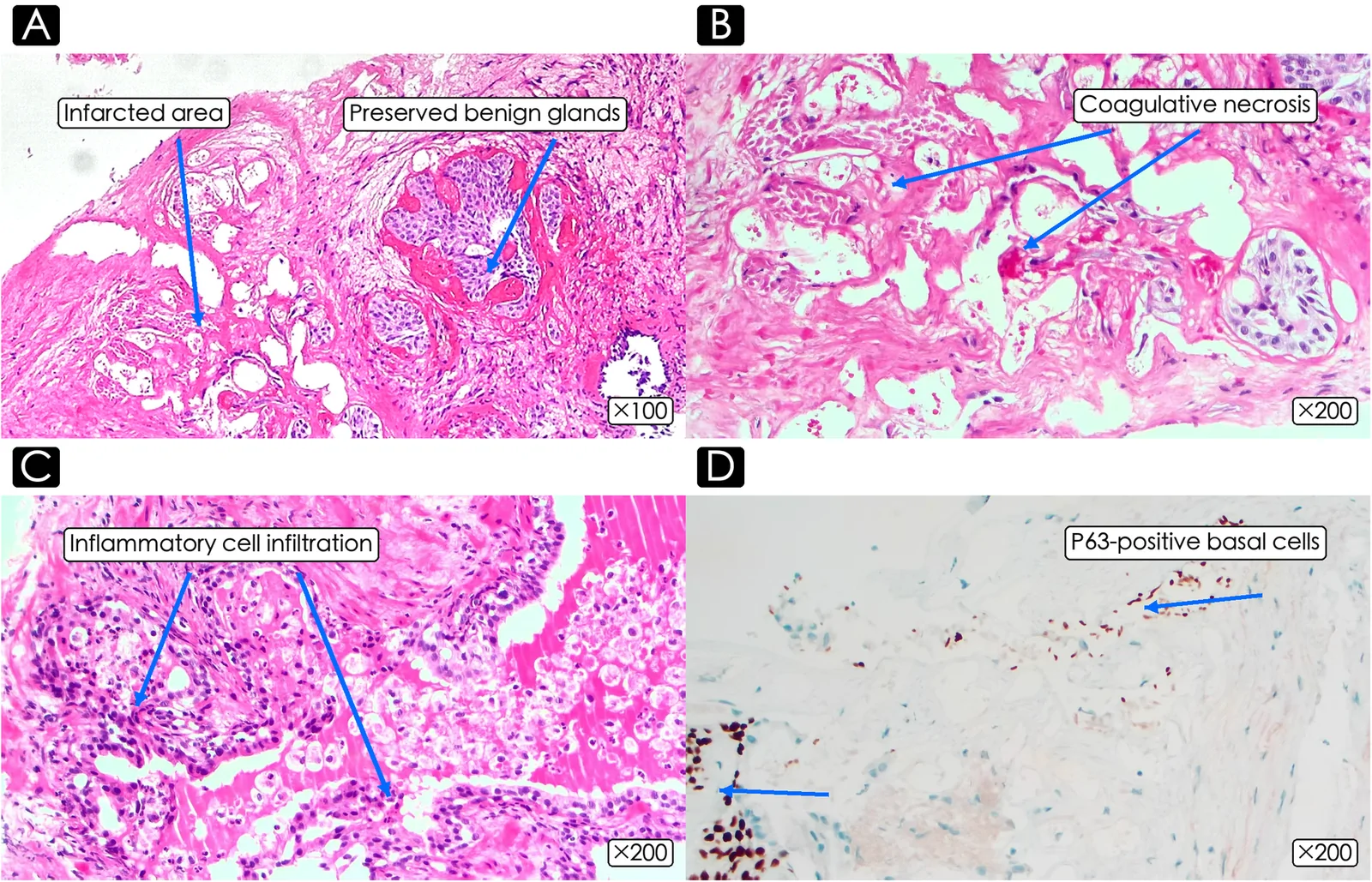
Histopathological and immunohistochemical findings of prostatic infarction. **(A)** H&E staining of the biopsy specimen showing an infarcted area and preserved benign glands (×100). **(B)** H&E staining showing coagulative necrosis with residual ghost-like glandular architecture (×200). **(C)** H&E staining of peri-infarct tissue showing inflammatory cell infiltration (×200). **(D)** P63 immunohistochemistry showing preserved P63-positive basal cells in residual glands (×200), supporting a benign lesion rather than prostatic adenocarcinoma.

## Therapeutic intervention

Because the patient had persistent lower urinary tract symptoms, urinary retention requiring catheterization, a large pre-catheterization residual volume, and imaging evidence of benign prostatic enlargement, transurethral resection of the prostate (TURP) was performed under general anesthesia on April 11, 2025. The procedure was intended to relieve bladder outlet obstruction and obtain additional tissue for pathological confirmation. Intraoperatively, a purple-red nodular lesion measuring approximately 20 × 15 mm with clear margins was identified in the left prostatic lobe (Figure 1D).

Postoperative histopathological examination of the TURP specimen again confirmed BPH with focal basal cell hyperplasia and infarction (Figures 2B,C). The infarcted focus measured approximately 20 × 15 × 4 mm, whereas the overall prostate specimen measured 50 × 40 × 35 mm. The concordant biopsy and resection findings established the final diagnosis of focal prostatic infarction associated with BPH rather than malignancy.

## Follow-up and outcomes

The postoperative course was uneventful. After TURP, the patient reported marked improvement in voiding symptoms, including smooth voiding, a stronger urinary stream, and nocturia reduced to 0–1 times per night. At 1 month after TURP, total PSA decreased from 31.858 ng/mL to 2.34 ng/mL, with free PSA of 1.23 ng/mL and a free-to-total PSA ratio of 0.53. At 3 months, total PSA remained low at 2.45 ng/mL, with free PSA of 1.25 ng/mL and a free-to-total PSA ratio of 0.51. At 1 year after surgery, telephone follow-up showed that the patient continued to void smoothly and reported nocturia 0–1 times per night. On August 10, 2026, approximately 16 months after TURP, repeat testing showed a total PSA of 1.234 ng/mL and free PSA of 0.421 ng/mL, with a free-to-total PSA ratio of 0.34, confirming sustained biochemical improvement.

## Discussion

The clinical importance of this case lies in the marked PSA elevation that prompted concern for prostate cancer, whereas the final diagnosis was a benign ischemic lesion. Although the free-to-total PSA ratio was very low (0.04), its diagnostic significance at a total PSA level of 31.858 ng/mL is uncertain because this value lies outside the range in which the ratio has been most extensively validated. To our knowledge, a very low free-to-total PSA ratio has not been established as a characteristic biochemical feature of prostatic infarction; therefore, the ratio of 0.04 in this case should not be interpreted as being specific to infarction. In routine practice, when prostate cancer is not confirmed, benign prostatic hyperplasia, prostatitis, and prostatic abscess are often considered as alternative explanations for PSA elevation. Prostatic infarction should therefore also be considered in the differential diagnosis.

The marked PSA elevation in this case was likely multifactorial. Prostatic infarction itself has been associated with elevated serum PSA (11). Acute urinary retention can also transiently increase serum PSA, with this effect potentially persisting for approximately 2 weeks (12). In our patient, PSA was measured approximately 6 days after catheterization for acute urinary retention, making retention-related PSA elevation an important potential confounder. Available evidence suggests that nontraumatic urethral catheterization alone has only a limited effect on PSA levels (13). Furthermore, the postoperative PSA decline cannot be attributed solely to resolution of prostatic infarction, because TURP itself substantially reduces PSA through removal of benign prostatic tissue (14). Therefore, prostatic infarction should be regarded as a plausible contributor to the PSA abnormality rather than its sole proven cause. Although total PSA decreased by approximately 92.7% at 1 month after TURP, infarction-specific PSA decline kinetics have not been established. Therefore, the magnitude and rate of PSA decline in this case should be interpreted descriptively rather than as a diagnostic feature of prostatic infarction.

There was no documented history of atrial fibrillation or other known systemic embolic source. Although a systemic embolic mechanism cannot be completely excluded, the combination of long-standing hypertension, marked prostatic enlargement (estimated volume, approximately 77 mL), and acute urinary retention with a pre-catheterization PVR of approximately 430 mL suggests that both vascular risk and local hemodynamic factors may have contributed to prostatic ischemia in this patient. These factors may have increased susceptibility to focal infarction; however, a definitive causal mechanism cannot be established from this single case.

Table 2 summarizes representative reports and the present case. Compared with previous reports, the present case is notable for marked PSA elevation with a very low free-to-total PSA ratio, PI-RADS 1 MRI findings, a focal CEUS perfusion defect, dual pathological confirmation by biopsy and TURP, immunohistochemistry, and sustained low postoperative PSA levels during approximately 16 months of follow-up.

**Table 2:** Concise comparison of representative reports of prostatic infarction and the present case.

Reference	Patients/Clinical presentation	PSA	Imaging	Pathological diagnosis	Key relevance
Rogers, 1947 (4)	Early clinicopathological report	Pre-PSA era	Not reported	Prostatic infarction	Early report establishing prostatic infarction as a pathological entity
Milord et al., 2000 (1)	15 patients; mean age 71 years (57–84); acute urinary retention in 4 of 12 patients with available clinical data	Three patients without urinary retention had PSA increases of 199, 219, and 287 ng/mL	Not reported	Prostatic infarction on needle biopsy	Demonstrated that prostatic infarction is rare on routine needle biopsy and may cause marked PSA elevation and diagnostic confusion
Kiran, 2001 (2)	Five patients with acute urinary retention	Markedly elevated PSA	Not reported	Prostatic infarction with chronic prostatitis	Suggested that prostatic infarction, urinary retention, and prostatitis may jointly contribute to marked PSA elevation
Present case	69-year-old man with hypertension, BPH, progressive LUTS, acute urinary retention, and a pre-catheterization PVR of approximately 430 mL	Total PSA 31.858 ng/mL; free-to-total PSA ratio 0.04	PI-RADS 1 MRI; focal CEUS perfusion defect	Biopsy and TURP confirmed focal prostatic infarction with BPH; no malignancy	Pre-biopsy CEUS localization, dual pathological confirmation, and sustained PSA decline at approximately 16 months

BPH, benign prostatic hyperplasia; CEUS, contrast-enhanced ultrasound; LUTS, lower urinary tract symptoms; MRI, magnetic resonance imaging; PSA, prostate-specific antigen; PVR, postvoid residual urine volume; TURP, transurethral resection of the prostate.

The discordance between markedly elevated PSA and PI-RADS 1 MRI findings was central to the diagnostic challenge. Although a PI-RADS score of 1 indicates a very low likelihood of clinically significant cancer, a non-suspicious MRI does not eliminate cancer, particularly when PSA is markedly abnormal and symptoms are progressing (8). In the present case, the combination of markedly elevated PSA, a very low free-to-total PSA ratio, urinary retention, and a focal CEUS perfusion abnormality supported further tissue evaluation. CEUS added functional information by identifying a focal perfusion abnormality. Ischemic or necrotic tissue may appear as a low-enhancement or non-enhancing defect, but this pattern is not specific; abscess, granulomatous inflammation, necrotic tumor, and other focal processes can also alter perfusion. Thus, CEUS was useful as a localization aid for biopsy rather than as a definitive diagnostic test (9). The absence of a palpable abnormality on digital rectal examination despite the imaging-detected lesion may partly reflect its transition-zone location and represents an additional teaching point of this case.

From a clinical perspective, this case does not support routine CEUS for all patients with elevated PSA and non-suspicious MRI findings. However, when marked PSA elevation is discordant with a low-suspicion MRI, particularly in the presence of urinary retention, vascular risk factors, or focal abnormalities on conventional ultrasound, CEUS may provide complementary perfusion information and help identify regions for targeted sampling. Prostatic infarction should also be considered among the benign differential diagnoses before biopsy in such discordant cases, although tissue confirmation remains necessary when malignancy cannot be confidently excluded. Thus, CEUS may serve as an adjunct rather than a replacement for MRI or histopathological evaluation.

Histopathology and immunohistochemistry resolved the diagnostic dilemma. Infarcted tissue is typically characterized by coagulative necrosis, ghost-like glandular outlines, hemorrhage, inflammatory infiltration, and reparative change (1). In the present case, preserved basal cells highlighted by P63 and CK5/6, negative P504s staining, and a low Ki-67 index argued against adenocarcinoma. TURP also served both therapeutic and confirmatory purposes by relieving obstruction and providing additional tissue for verification.

The main strengths of this case are the complete diagnostic sequence linking biochemical findings, MRI, CEUS, systematic and targeted biopsy, TURP pathology, immunohistochemistry, and biochemical follow-up extending to approximately 16 months after TURP, during which PSA remained low. Nevertheless, the postoperative PSA decline is not specific for prostatic infarction because resolution of urinary retention and removal of benign prostatic tissue by TURP may also have contributed. This single case cannot establish the diagnostic performance of CEUS or the incidence of prostatic infarction. The findings from this single case should not be generalized to all patients with elevated PSA and non-suspicious MRI findings.

Important limitations include the absence of baseline IPSS and formal uroflowmetry, which limited quantitative assessment of changes in lower urinary tract symptoms and urinary flow; the measurement of PSA during the post-retention period; the lack of quantitative ADC data; and the absence of quantitative CEUS perfusion parameters. The patient also had elevated aminotransferase levels in the setting of long-term heavy alcohol consumption; however, no definite alcohol-related liver disease had been diagnosed. The potential influence of chronic alcohol use and hepatic dysfunction on PSA kinetics in this case remains uncertain.

Prostatic infarction should remain in the differential diagnosis in elderly men with vascular risk factors, BPH, urinary retention, marked PSA elevation, and laboratory-imaging discordance. Standard prostate-cancer evaluation, tissue confirmation, and follow-up PSA surveillance remain essential.

## Patient perspective

At postoperative follow-up, the patient reported marked improvement in voiding symptoms, including smooth voiding and nocturia reduced to 0–1 times per night. At 1 year after surgery, he continued to report smooth voiding without a sense of obstruction.

## Data Availability

The original contributions presented in the study are included in the article/Supplementary Material, further inquiries can be directed to the corresponding author.
